# Chia gum-gelatin-based encapsulation of chia sprouts phenolic compounds enhanced storage stability, bioavailability, antioxidant, antidiabetic, and antibacterial properties

**DOI:** 10.1038/s41598-024-71913-2

**Published:** 2024-09-25

**Authors:** Azza M. Abdel-Aty, Amal Z. Barakat, Roqaya I. Bassuiny, Saleh A. Mohamed

**Affiliations:** Molecular Biology Department, National Research Centre, Dokki, Cairo, Egypt

**Keywords:** Chia sprouts, Encapsulation, Phenolic compounds, Stability, Functional properties, Biochemistry, Biological techniques, Biotechnology

## Abstract

Chia seeds are currently gaining popularity as functional and healthy foods. The developed chia 7-day sprout phenolic extract (CSP) is an abundant supply of highly concentrated antioxidant phenolic compounds with health-promoting and antibacterial properties. The easy destruction against different environmental changes and low bioavailability of these phenolic compounds are the main limitations of their applications/utilization. This study aims to microencapsulate the phenolic compounds of developed CSP for use as valuable functional food additives. Three microcapsules were prepared using coating materials, chia gum (CG), gelatin (G), and their mixture (CG/G) via the freeze-drying technique. The prepared CG-, CG/G-, and G-microcapsules demonstrated high encapsulation efficiency percentages of 97.0, 98.1, and 94.5%, respectively. They retained most of the CSP-phenolics (91.4–97.2%) and increased total antioxidant activity (108–127.1%). The prepared microcapsules released more CSP-phenolic compounds into the simulated intestinal stage (70–82%) than the gastric stage (15–24%), demonstrating that the coating materials enhance protection during the gastric stage. The produced microcapsules exhibited higher storage stability at 40 °C for 60 days than the non-capsulated CSP, indicating that the encapsulation provided enhanced stability. The prepared microcapsules microstructures showed uniform, smoother surfaces, and hidden micropores compared to their coating material microstructures. In addition, the connection between the functional groups of coating materials and CSP-phenolic compounds was demonstrated by FTIR analysis. The prepared CG-, CG/G-, and G-microcapsules can perfectly inhibit the α-amylase and α-glucosidase activities by 65, 68, 60 and 74, 78, and 70%, respectively, compared to CSP (54, and 66%). The three prepared microcapsules displayed better antibacterial with low MBC values (0.36–0.68 mg ml^−1^) compared to CSP (0.53–0.74 mg ml^−1^). The prepared CSP microcapsules can be incorporated into various food products to enhance their antioxidant, antidiabetic, and antibacterial properties.

## Introduction

Natural phenolic compounds are highly valuable plant byproducts that efficiently scavenge free radical oxygen species and make complexes with various polymers. These abilities employ phenolic compounds in health promotion, disease prevention, and disease treatment, like diabetes, inflammation, cancer, antimicrobial, antioxidant, and anti-snake^1–6^. However, the phenolic compounds are unstable against different environmental changes, which is challenging when used in food processing and/or in the human diet due to their reduced antioxidant and beneficial health properties. Additionally, many of these compounds have offensive tastes and odors, which limit their application in food or oral therapies^7^.

Among the existing stabilization methods, encapsulation is a useful technology that protects phenolic compounds by covering them in a resistant layer, such as a polymeric matrix, and solving phenolic compound problems. Encapsulation is the process of trapping a core material (such as bioactive phenolic compounds) within other immiscible materials (wall materials). The encapsulation method covers up the unpleasant odor, taste, and color of the phenolic compounds while also extending their shelf life, stabilizing them in the digestive tract or during food processing, and improving their bioavailability and bioactivity^8,9^.

Choosing the suitable protective wall material is based on the required properties and the use of the product. Polysaccharides, and proteins, are widely used as wall materials to protect and deliver bioactive compounds^10^. Chia gum (CG) was isolated and purified from chia seeds. CG is a hydrophilic heteropolysaccharide with a high concentration of uronic acids, which give the macromolecule anionic properties. It possesses high viscosity, thermal stability, superior emulsifying and gelling properties, potential biodegradability, high hydration capacity, and low cost, and is non-toxic^11^. Thus, CG is a promising polymer for producing a biopolymeric microcapsule^12^. Gelatin (G) is one of the most widely employed animal proteins. It is a biodegradable protein derived from collagen. It remains the first material employed as a wall in many encapsulation applications due to its excellent emulsifying, gelling, thickening, water-soluble nature, high stabilizing, and strong crosslinking action via its main amino groups as well as safe, high nutritional value, easily digested^13,14^.

Recently, we demonstrated that the germination of the chia seeds enhanced/maximized their total phenolic and flavonoid levels (6.4 and 11.5 folds, respectively) and antioxidant activity (10-, 17-, and 29-folds using DPPH, ABTS, and PMC antioxidant methods, respectively). Based on HPLC investigation, 12 phenolic acids, and 5 flavonoids were demonstrated in chia 7-day sprouts with higher concentrations than the chia dry seeds. Two new phenolic compounds were also observed: *p*-coumaric acid and kaempferol in chia 7-day sprouts. Moreover, Egyptian chia 7-day sprouts exhibited potent antibacterial action against human pathogenic bacteria^15^. Therefore, chia 7-day sprout phenolic extract (CSP) is considered an abundant supply of antioxidant phenolic compounds and could be utilized as a functional food to prevent or treat many diseases, as well as an antibacterial agent. However, no studies were reported on the microencapsulation of the CSP-phenolic compounds. Therefore, the current study aims to produce new microcapsules of CSP, as core material, using wall materials G and CG, both alone and in combination to evaluate their properties/impacts. These developed microcapsules introduce powerful natural CSP-antioxidant compounds in the food and pharmaceutical industries.

## Materials and methods

### Seven-day chia sprout germination

The Egyptian chia seeds were provided and identified by the Botany Department of the Agriculture Research Centre, Giza, Egypt. According to Abdel-Aty et al.^15^, a significant amount of chia 7-day sprouts germinated. Briefly, chia seeds were surface-sterilized and put on a plastic plate with wet tissue paper. Chia seeds were germinated at room temperature and in the dark. They watered daily and the sprouting seeds were collected seven days later and oven-dried overnight at 45 °C before being crushed. The chia plant-collecting material and its conducted experiments complied with all relevant institutional, national, and international guidelines and legislation.

### Chia-sprouts phenolic-rich extract (CSP) preparation

Chia-7-day sprout powder was extracted in 80% methanol under continuous overnight shaking (at 200 rpm). Filter paper (Whatman 1) was used to filter the mixture. The obtained filtrate/extract (CSP) was concentrated using a rotary evaporator at 45 °C, freeze-dried at − 55 °C, and kept at − 20 °C.

### Chia seed gum preparation

According to Abdel-Aty et al.^9^ procedure, chia dry seeds were soaked in distilled water at a ratio of 1:20 (Seed: Water) (w/v) for three hours at 45 °C. Filtration was employed to separate the resulting mucilage. One volume of mucilage was mixed with three volumes of ethanol, and the resulting precipitate was collected, dried, and crushed.

### Encapsulation of CSP

Gelatin (G) and prepared chia gum (CG) were employed as coating materials to encapsulate the CSP. A hundred mg of either G or CG was separately dispersed in a hundred ml of distilled water at 500 rpm stirring and at 40 °C for 1 h before the encapsulating procedure. The CSP was combined with the coating materials at three distinct cores in the following ratios CG: CSP (10:1), CG + G: CSP (5 + 5:1), and G: CSP (10:1). For one hour, the mixtures were stirred at 1500 rpm to achieve a uniform dispersion of wall materials with CSP. Each prepared mixture was freeze-dried at − 55 °C after being frozen at − 80 °C. The prepared microcapsules were homogenized in a mortar and pestle and stored in an airtight container at 4 °C.

### Total phenolic content and total antioxidant activity

Ten mg of CSP or the prepared microcapsules were mixed in 1 ml of ethanol, acetic acid, and water at a 50:8:42 ratio for 1 min. After vortex for 1 min, the resulting mixture was filtered through a 0.5 µm filter^16^.

For measuring total phenolic content (TPC), 50 µl of each prepared microcapsule-filtrate or CSP was mixed with Folin-Ciocalteu reagent (100 µl) and distilled water (850 µl) and incubated for 5 min at room temperature according to Veliogluet al.^17^ method. Following adding 500 µl of sodium carbonate (20%), the mixture was incubated for 30 min, and the absorbance at 750 nm was determined. The TPC was expressed as mg gallic acid equivalent (GAE).

For measuring total antioxidant activity (TAA), 900 µl of 0.1 mM DPPH dissolved in methanol and 100 µl of each prepared microcapsule-filtrate or CSP were incubated for 30 min in the dark at room temperature, and the absorbance at 517 nm was determined. The antioxidant activity was measured using Eq. 1:

1 $$ {\text{DPPH scavenging }}\% \, = \, \left( {{\text{O}}.{\text{D}}.{\text{ control }}{-}{\text{O}}.{\text{D}}.{\text{ sample}}} \right)/{\text{O}}.{\text{D}}.{\text{ control}} \times {1}00 $$

The TAA was expressed as Trolox equivalent (TE).

### Surface phenolic content (SPC)

Ten mg of the prepared microcapsules were mixed in one ml of ethanol and methanol at a 1:1 ratio for one minute. After vortex for one minute, the resulting mixture was filtered^18^. The SPC of each microcapsule filtrate was measured as described above by the Folin-Ciocalteu method.

### Encapsulation efficiency

According to Cilek et al.^18^, the encapsulation efficiency (EE) of the obtained microcapsules was calculated using Eq. (2):

2 $$ {\text{EE }}\% \, = \, \left( {{\text{TPC}} - {\text{SPC}}/{\text{TPC}}} \right) \, \times { 1}00 $$

TPC: Total phenolic content of the prepared microcapsule.

SPC: Surface phenolic content of the prepared microcapsule.

### Gastro-intestinal digestion

The TPC released from the produced microcapsules was measured in two different conditions: simulated gastric fluid (SGF) and simulated intestinal fluid (SIF). Each microcapsule was distributed in 10 ml SGF-solution (0.3 mM NaCl, 4 mg pepsin, and 0.1 M HCL at pH 2.0) and shaken at 100 rpm for two hours at 37 °C. After adjusting the pH of the mixture to 7.3, the pancreatin (SIF-solution) was added and also shaken at 100 rpm for two hours at 37 °C^19^. Following the end of each step of digestion, the sample was neutralized using a 100 mM NaHCO_3_ solution, and centrifuged for 10 min at 5000 rpm, and the Folin-Ciocalteu method was used to quantify the release phenolic content. The digestibility index was determined using Eq. (3):

3 $$ {\text{Digestibility index }} = \, \left( {{\text{PC}}_{{{\text{Release}}}} /{\text{TPC}}} \right) \, \times { 1}00 $$

PC _Release_: Phenolic content released from the microcapsule.

TPC: Total phenolic content found in microcapsule.

### Stability during storage

The CSP powder and each prepared microcapsule were sealed individually in brown vials (15 mg/vial) and stored at 40 °C for 60 days. Samples were checked every fifteen days, and the TPC and TAA were determined as mentioned above.

### Physicochemical properties of microcapsules

#### Moisture

Each microcapsule (50 mg) was dried at 105 °C for 24 h and the difference between weights (mg) of each microencapsulate pre- and post-drying was investigated^20^.

#### Solubility

Each microcapsule (100 mg) was combined with 10 ml of deionized water, vortexed for 1 min, stored for 30 min at 37 °C, and centrifuged for 5 min at 4000 rpm. Each supernatant was dried at 105 °C for 24 h. The difference between the initial weight and the supernatant weight was used to measure the solubility (%)^20^.

#### Swelling

Each microencapsulate (50 mg) was submerged in distilled water for 3 h at 28 °C. The samples were vacuum-filtered and water excess was removed by filter paper, and the weight was then measured again^21^. The difference between weights (mg) of each microcapsule pre- and post-swelling was calculated.

#### Surface morphology

The microstructure of the coating materials (G and CG-gum) and the prepared microcapsules was examined using a scanning electron microscope (SEM) (FE-SEM, Quanta FEG 250) at an accelerating voltage of 20 kV.

#### FT-IR

The coating materials (G and CG-gum) and the prepared microcapsules were exposed to FT-IR analysis using Bruker ALPHA-FT-IR-Spectrometer. The scanning wave ranged from 400 to 4000 cm^−1^.

#### Antidiabetic activity

For α-amylase inhibition, five U of pancreatic α-amylase were pre-incubated with 20 mM sodium phosphate buffer (pH 7.2) and CSP or each prepared microcapsule for 5 min at 37 °C. Next, 0.1 ml of starch (1.0%) was added and stored for 30 min at 37 °C. Following the addition of 500 µl of dinitro-salicylic reagent and boiling for 10 min, the absorbance was read at 540 nm^22^. Acarbose was used as a standard. The inhibition % was calculated according to Eq. (4).

4 $$ {\text{Inhibition }}\% = \, \left( {{\text{O}}.{\text{D}}.{\text{ control }}{-}{\text{ O}}.{\text{D}}.{\text{ test}}} \right)/{\text{O}}.{\text{D}}.{\text{ control }} \times { 1}00 $$

For α-glucosidase inhibition, one U of α-glucosidase was pre-incubated with 20 mM sodium phosphate buffer (pH 6.8) and CSP or each prepared microcapsule for 5 min at 37 °C. Following the addition of 2 mM *p*-nitrophenyl-α-glucopyranoside the incubation for 15 min at 37 °C was occurred. The absorbance was read at 405 nm^23^. Acarbose was used as a standard. The inhibition % was calculated according to Eq. (5).

5 $$ {\text{Inhibition }}\% = \, \left( {{\text{O}}.{\text{D}}.{\text{ control }}{-}{\text{ O}}.{\text{D}}.{\text{ test}}} \right)/{\text{O}}.{\text{D}}.{\text{ control }} \times { 1}00 $$

#### Antibacterial activity

The antibacterial efficacy of CSP and the prepared microcapsules against gram-negative and gram-positive human pathogenic bacterial strains, *Escherichia coli* O157-H7 ATCC 51659 and *Staphylococcus aureus* ATCC 13565, respectively, were evaluated using broth dilution and colony counting strategies^24^. Briefly, each examined bacterial strain was grown in falcon tubes filled with Muller-Hinton broth that was mixed with different doses of either CSP or the prepared microcapsules. The incubation took place under shaking for 18 ± 1.0 h at 37 °C. Each falcon tube solution was cultured on Mueller Hinton agar plates and incubated at 37 °C for 18 ± 1.0 h before the number of bacterial colonies in each was counted. The minimum bactericidal concentration (MBC), which is the minimal concentration that kills 99.9% or more of the initial inoculation, was used to assess the antibacterial activity.

### Statistical analysis

The statistical analysis was calculated using a one-way ANOVA. The data were considered as means ± S.D. (n = 4).

## Results and discussion

### Total phenolic content, total antioxidant activity, and encapsulation efficiency

The levels of total phenolic content (TPC) and total antioxidant activity (TAA) of CSP in the prepared microcapsules are an important/essential part of the functional food, cosmetics, and pharmaceutical industries. They are used to ensure the quality of the product, its functional value, and its optimal preservation, which is the main purpose of this study. Table 1 shows the TPC, and TAA of the CSP and prepared microcapsules and the encapsulation efficiency (EE). The prepared microcapsules displayed slightly lower TPC (ranging from 45.7 to 48.6 mg GAE) than the TPC of the non-encapsulated CSP (50.0 mg GAE). This reduction in TPC is possibly due to the conditions of the encapsulation process and drying technique which might not fully keep the phenolics in the microcapsules. This finding was consistent with previous studies that used the freeze-drying method to encapsulate phenolic compounds^9,25,26^. However, the prepared microcapsules demonstrated higher TAA (ranging from 46.2 to 54.4 µmol TE) than the TAA of the unprocessed CSP extract (42.8 µmol TE). This finding may be explained by increased concentrations of certain phenolic antioxidant compounds, after the microencapsulation procedure, which are more sensitive to scavenging DPPH radicals. The quercetin-3-glucoside level was significantly increased in gum Arabic microcapsule of sour cherry phenolic extract than in non-capsulated extract^27^. Catechin concentration was also increased after encapsulation of blackberry phenolic extract compared to the initial blackberry extract^28^. The antioxidant activity of the flour-encapsulated was also higher than that of non-capsulated flour^29^. The garden cress sprouts-phenolic extract recorded higher total antioxidant activity in the garden cress gum microcapsule than in the non-capsulated phenolic extract^9^. Besides, chia gum or gelatin as coating materials may have antioxidant activity providing excess antioxidant activity. Some studies demonstrated the strong antioxidant activity of many gums and gelatin^30–32^.

**Table 1:** Total phenolic content (TPC), surface phenolic content (SPC), encapsulation efficiency (EE), and total antioxidant activity (TAA) of freeze-dried chia 7-day sprout phenolic extract encapsulated with varied wall materials used in various ratios.

Sample	Wall: CSP ratio	TPC mg GAE	SPC mg GAE	EE (%)	TAA µmol TE Using DPPH	TAA retention %
CSP	–	50.0 ± 2.0^a^	–	–	42.8 ± 2.1^a^	100.0^a^
CG	10:1	48.5 ± 1.2^a^	1.5 ± 0.07^a^	97.0^a^	54.4 ± 1.7^b^	127.1^b^
CG + G	5 + 5:1	48.6 ± 1.3^a^	0.9 ± 0.04^b^	98.1^b^	52.8 ± 2.0^b^	123.3^c^
G	10:1	45.7 ± 1.1^b^	2.5 ± 0.08^c^	94.5^c^	46.2 ± 1.0^c^	108.0^d^

Results are provided as means ± SD (n = 4); values in the same column with different superscripts are significantly different at (*p* < 0.01).

GAE, Gallic acid equivalent; TE, Trolox equivalent; CSP, Chia 7-day sprout phenolic extract; G, Gelatin; CG, Chia Gum.

Evaluation of encapsulation efficiency (EE%) is an important indicator/analysis for assessing the success of the encapsulation process. This analysis makes it possible to determine the level of protection provided to the phenolic compounds and their functional properties. The obtained EE% values are 97.0, 98.1, and 94.5% for CG-, CG/G- and G-microcapsules, respectively as seen in Table 1. The mixture of CG and G coating materials exhibited a greater EE% (98.1%). This result demonstrated that the selected coating material is a key factor for phenolic compound encapsulation. The higher emulsifying properties and heteropolysaccharide nature (hydrophobic and hydrophilic properties) of the CG-gum, as well as the gelling and higher emulsifying features of the G, entrap the phenolic compounds, forming a three-dimensional network that may be responsible for higher retention of the CSP-phenolic compounds. The obtained results were found higher than some other previous studies. The EE% of nine developed microcapsules ranged from 72.70 to 83.65%, where the coating materials were gelatin and acacia gum and the core material was anthocyanin^33^. Also, lower EE% of microcapsules developed from the gelatin and gum Arabic coating materials, and ascorbic acid core material^34^. Similar to our findings, the gum Arabic and maltodextrin mixture displayed great EE% ranging from 93 to 98.3% when used to encapsulate some bioactive compounds^26^. Furthermore, the garden cress sprouts-phenolic extract recorded a higher EE% in the garden cress gum microcapsule (98.2%) among eight developed microcapsules, with EE% ranging from 80.3 to 96.5%^9^. These variations in EE% values may be due to differences in coating materials, ratios, core materials, drying methods, and encapsulation process conditions.

### Gastrointestinal-digestion

**Fig. 1:**
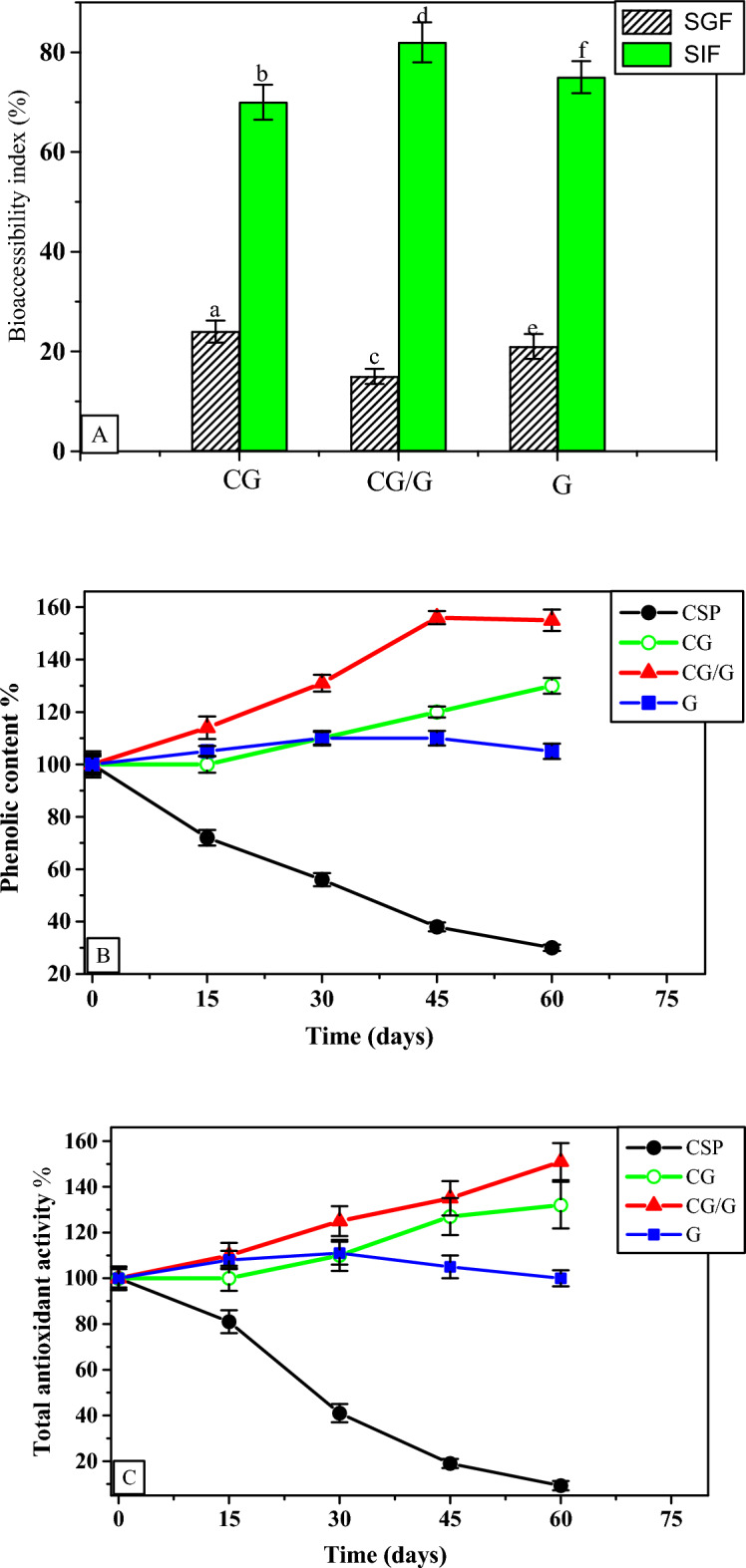
(**A**) Release of TPC from the prepared microcapsules in simulated gastric fluid (SGF) and simulated intestine fluid (SIF), (**B** and **C**) storage stability of the TPC and TAA of the CSP and the prepared microcapsules at 40 °C for 60 days. TPC, total phenolic content; TAA, total antioxidant activity; CSP, Chia 7-day sprout phenolic extract; CG, Chia gum-microcapsule; CG/G, Chia gum-gelatin microcapsule and G, gelatin-microcapsule. Values are presented as means ± SD (n = 4); different letters are statistically different at (*P* < 0.01).

The release of TPC from the prepared microcapsules was analyzed using the simulated gastrointestinal pH conditions to track how the TPC is delivered at specific points where absorption occurs. Figure 1A shows the digestibility of the prepared microcapsules in SGF and SIF for 2 h at 37 °C. In SGF, the release of TPC from the CG-, G-, and CG/G-microcapsules was significantly reduced, with digestibility index values of 24, 21, and 15%, respectively. However, in the SIF, the release of TPC from the CG-, G-, and CG/G-microcapsules was significantly increased, with digestibility index values of 70, 75, and 82%, respectively. All the prepared microcapsules exhibited greater digestibility values in SIF than within SGF. This observation demonstrates that the gelatin and chia gum and their combination provide greater protection for TPC in the gastric phase and improve their accessibility in the intestinal phase. The reduced TPC level in the gastric condition could be caused by little phenolic compounds released at low pH (2.0) from the prepared microcapsules and/or by little SGF entering the microcapsules through their surface/coating material, suggesting that the chia gum, gelatin, and particularly their combination are suitable coats for protection and delivery of the CSP phenolic compounds. Wang et al.^35^ reported that the gelatin digestion rate and its antioxidant activity in the SIF significantly increased compared to the SGF, confirming that more peptide bonds were hydrolyzed by pancreatin. The CG-, G-, and CG/G-microcapsules can therefore be added to various food products. Similar results were previously reported for the releasing of moringa extract microcapsules using maltodextrin and pectin^36^, bioactive phenolic compound microcapsules using maltodextrin and Arabic gum^26^, and garden cress sprout extract microcapsules using garden cress gum^9^.

### Storage stability

Changes in the TPC and TAA of the prepared microencapsulated powders compared to non-encapsulated CSP stored at 40 °C for 60 days are presented in Fig. 1B and C. The TPC and TAA of the non-encapsulated CSP extract were gradually reduced to 70 and 90%, respectively, after 60 days of incubation at 40 °C. This finding demonstrated that TPC and TAA in CSP are not protected and are easily accessible to interact with the surrounding environment. However, retention of the TPC and TAA remained constant (~ 100%) in the prepared G-microcapsule, demonstrating that the gelatin-wall material can perfectly protect the CSP extract bioactivity during 60 days of incubation at 40 °C. Interestingly, the TPC and TAA of CG- and CG/G-microcapsules gradually increased by 130, 155, and 132, 151%, respectively, after 60 days of incubation at 40 °C. The increase in TPC and TAA in CG- and CG/G-microcapsules may be due to the influence of the higher temperature on the hydrolysis of phenolic glycosides and the generation of greater amounts of free phenolics, which are highly sensitive to DPPH free radicals and the Folin-Ciocalteu reagent. At the same time, these generated free phenolic compounds were protected by wall materials of microcapsules. Similarly, the TPC of the purple cactus pear extract increased nearly twofold in the three microencapsulates after 50 days of incubation at 60 °C^37^. The retention of the TPC remained constant in all maltodextrin and Arabic gum capsules that were produced for banana-peel extract after 30 days of incubation at 40 °C^20^. On the contrary, for microencapsulated anthocyanin from black rice bran using a double emulsion complex (gelatin and acacia gum), the microcapsules were degraded by 15–25% after 7 weeks of storage under 37 °C^33^. For microencapsulated anthocyanin from black raspberry using a double emulsion complex (gelatin and Arabic gum), the microcapsules maintained ~ 13–24% of the initial concentration of anthocyanin after 60 days of storage under 37 °C^13^. From the above findings and discussion, the storage stability was significantly dependent on the wall material properties such as embedding capacity and core retention ability. Consequently, it can be determined that the CG and G effectively protect the antioxidant-phenolic compounds of the CSP and the three prepared microencapsulates are suitable for long-term preservation even under difficult storage conditions.

### Physical properties of the prepared microcapsules

#### Moisture, solubility, and swelling properties

The moisture, solubility, and swelling % of the prepared microcapsules are listed in Table 2. In microencapsulated products, moisture plays a significant role in determining flow state, storage stability, and viscosity in different food systems. The food industry targets moisture contents in the 1.0–7.0% range to ensure the stability of powder products during storage^38^. The prepared CG/G- and CG-microcapsule moisture contents (4.0 and 7.0%, respectively) fell within this range, while the prepared G-microcapsule showed slightly higher moisture content (10.0%), and all obtained results were close to those reported in several studies. Anthocyanin microcapsules using gelatin and acacia gum showed moisture contents ranging between 3.62 and 5.24%^33^. The black raspberry extract microcapsules using gelatin and Arabic gum also showed moisture contents ranging between 1.97 and 6.69%^13^.

**Table 2:** Moisture, solubility, and swelling % of the prepared CG, CG/G, G microencapsulates.

Microencapsulate	Moisture (%)	Solubility (%)	Swelling (%)
CG	7.0 ± 0.30^a^	33 ± 1.3^a^	115 ± 2.0^a^
CG/G	4.0 ± 0.12^b^	37 ± 1.8^b^	121 ± 4.0^b^
G	10.0 ± 0.24^c^	50 ± 2.0^c^	108 ± 3.3^a^

Values are provided as means ± SD (n = 4). Different letters in the same column are statistically different at (*P* < 0.01).

CG, Chia gum-microencapsulate; CG/G, Chia gum-gelatin microencapsulate; G, gelatin- microencapsulate.

The CG-microcapsule recorded a lower value of solubility of 33% compared to the CG/G- and G-microcapsules, which recorded 37 and 50%, respectively (Table 2). Reduced solubility allowed the core compounds to be released into the target gradually, slowly, and under control. It can be observed that a higher solubility of G-microcapsule may be due to the hydrophilic nature of gelatin. The mixing of G and CG reduced the solubility of the CG/G-microcapsule. Nine anthocyanin microcapsules using gelatin and acacia gum showed low solubility values of 11.94 to 15.92%^33^. The six black raspberry microcapsules using gelatin and Arabic gum showed solubility values of 15.04–30.32%^13^.

The swelling values of the prepared microcapsules were in the following order: CG/G < CG < G with 121, 115, and 108% (Table 2). The improved swelling property of the CG/G-microcapsule may be due to the gelling nature of the chia gum and gelatin, which swell when they absorb water. This property can conserve the structure and function of the core material. This explains the high ability of the prepared microcapsules to preserve the antioxidant phenolics of CS. Garden cress sprout extract microcapsule using garden cress gum showed improved swelling properties (141%) due to the gel nature of garden cress gum compared to maltodextrin microcapsule (102%)^9^.

#### SEM micromorphology

**Fig. 2:**
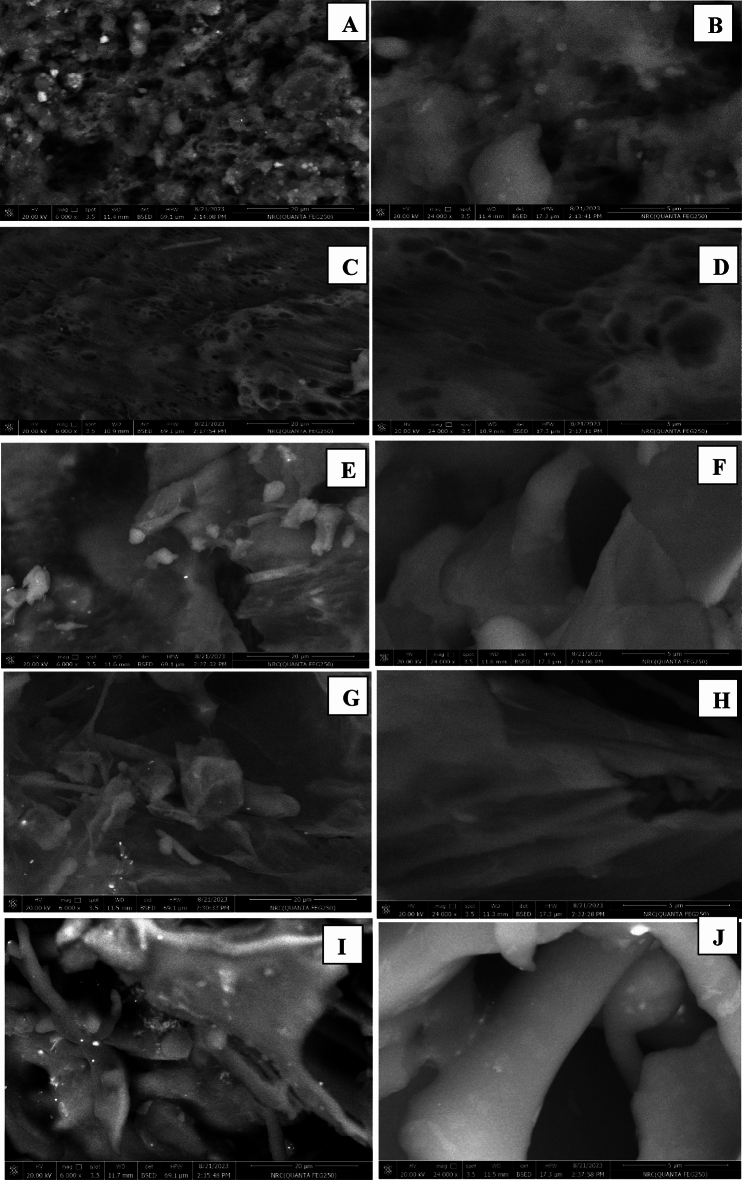
SEM images of pure chia gum (**A** and **B**) and gelatin (**C** and **D**) as coating materials, CG-microcapsule (**E** and **F**), CG/G-microcapsule (**G** and **H**), and G-microcapsule (**I** and **J**). Micro-images were taken at low and high magnifications. CG, Chia gum-microcapsule; CG/G, Chia gum-gelatin microcapsule; G, gelatin-microcapsule.

Figure 2 shows SEM images of wall materials, CG and G, and the three prepared microcapsules. Both wall materials, CG and G, have rough surfaces with a high degree of irregularity and micropores (Fig. 2A–D, respectively). Molecules with a rough surface can delay the release of core material and get trapped in their pores. The CG-, CG/G-, and G-microcapsules exhibited more uniform and smoother surfaces, having more regular shapes, and hidden micropores (Fig. 2E–J, respectively). The prepared microcapsules were observed as reservoirs where the wall material perfectly entrapped the core material. These structures allow the retention of more antioxidant-phenolic compounds and produce microencapsulates with a higher storage capacity. Furthermore, the CG/G-microcapsule looks more uniform and smoother surface than the CG- and G-microcapsules. Thus, the developed microcapsules are a better choice for preserving the antioxidant phenolics of CSP. The six black raspberry microcapsules using gelatin and Arabic gum showed similar uniform microstructures^13^. However, the maltodextrin and Arabic gum combination to encapsulate the pink pepper extract resulted in rougher particles with more irregular shapes^26^.

#### FTIR-spectrum analysis

**Fig. 3:**
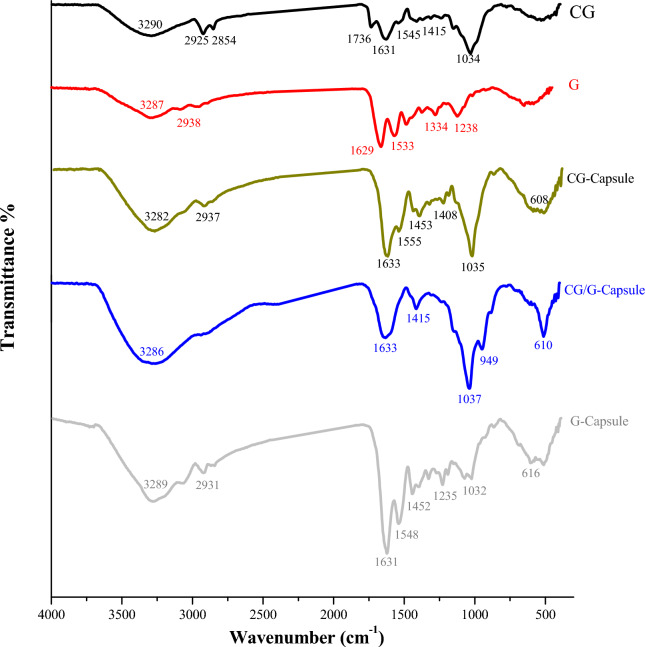
FTIR spectra of wall materials, chia gum, and gelatin, and the three prepared microcapsules. CG-, Chia gum-microcapsule; CG/G-, Chia gum-gelatin microcapsule and G-, gelatin-microcapsule.

Figure 3 displays the FTIR spectra of wall materials, CG and G, and the three prepared microcapsules. The CG spectrum showed a broad band of O–H stretching at 3290 cm^−1^ which makes up the basic structure of carbohydrates. The bands at 2925 and 2854 cm^−1^ were generated by the C–H aromatic ring stretching and the methyl group (CH_3_). The peaks at 1545 and 1412 cm^−1^ were linked to the uronic acid carboxyl group (–COO–). In addition, the negative carbonyl (C–O) stretching band was detected at 1034 cm^−1^. These identified bands of CG were the hydroxyl and carbonyl groups of gum and mucilage^9,39^. The G spectrum showed the typical N–H stretching of the amines and amide groups: amide A, amide B, amide I, and amide II at 3287, 2938, 1629, and 1533 cm^−1^, respectively. After the encapsulation process, the CG-microcapsule spectrum revealed vibrations of several O–H groups at 3282 cm^−1^ (high intense broadband), aromatic C–C stretching at 1408 cm^−1^, and CH_2_ rocking at 608 cm^−1^, all of which were linked to the structures of phenolic compounds^9^. Further, the O–H, C–H, –COO–, and C–O peaks were shifted from 3290, 2925, 1545, 1412, and 1034 to 3282, 2937, 1555, 1453 and 1035 cm^−1^, respectively, with high intensity. The G-microcapsule spectrum showed a highly intense broad band of several O–H groups at 3289 cm^−1^, aromatic C–C stretching at 1452 cm^−1^, and CH_2_ rocking at 616 cm^−1^ of the phenolic compound structures, as well as shifted gelatin amide A, amide B, amide I, and amide II at 3289, 2931, 1631, and 1548 cm^−1^, respectively, with high intensity. Also, the phenolic compound structures of CSP as several O–H groups at 3286 cm^−1^, and the CH_2_ band at 610 cm^−1^ have appeared in the CG/G-microcapsule spectra. In addition, the characteristic peaks of amide were shifted, intensified, and appeared at 3286, 1633, and 1415 cm^−1^. Moreover, the typical (C–O) stretching peak of CG was seen at 1037 cm^−1^. The three prepared microcapsule spectra showed the most functional groups of CSP-phenolic compounds and coating materials with obvious shifting, strong absorption, and broad intensities following the encapsulation process. This observation is related to the forming of several intermolecular hydrogen bonds between coating materials and CSP phenolic compounds. This is associated with the abundance of hydroxyl and carboxyl groups in CSP-phenolic compounds that increase the possibility of several intermolecular hydrogen bonds^40^. Such findings demonstrate the interaction between the functional groups of the wall materials and CSP phenolic compounds. In addition, the coating materials offered protection to the most functional CSP groups after the microencapsulation.

### Antidiabetic properties

**Fig. 4:**
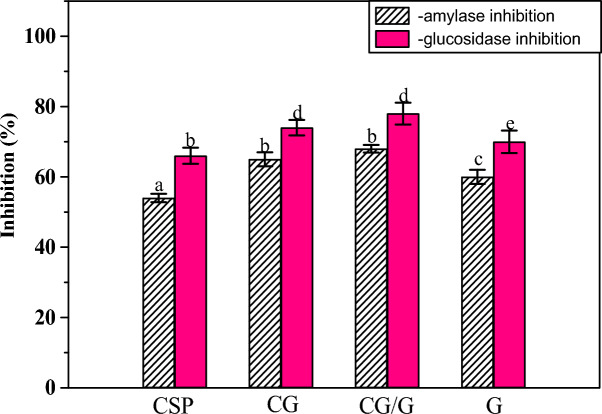
Antidiabetic potential of the three produced microcapsules compared to CSP extract. CSP, Chia 7-day sprout phenolic extract; CG, Chia gum-microcapsule; CG/G, Chia gum-gelatin microcapsule and G, gelatin-microcapsule. Values are presented as means ± SD (n = 4); different letters are statistically different at (*P* < 0.01).

Α-amylase and α-glucosidase natural inhibitors/phenolic compounds have become an appropriate therapy choice for reducing postprandial hyperglycemia. These compounds effectively reduce carbohydrate digestion, resulting in lower postprandial blood glucose. Therefore, the inhibition of α-glucosidase and α-amylase by unencapsulated CSP and the three produced microcapsules were evaluated as displayed in Fig. 4. It can be observed that the non-capsulated CSP extract was able to inhibit the α-amylase and α-glucosidase activities significantly by 54, and 66%, respectively. This is probably caused by the phenolic compounds attaching/blocking the active site of these enzymes (enzymes hydrolyze carbohydrates)^41^. Protocatechuic acid, rosmarinic acid, caffeic acid, catechin, and quercetin were the main polyphenols with high concentrations identified in CSP extract^15^. These phenolic compounds had the potential to inhibit α-amylase and α-glucosidase, improve insulin function, and reduce oxidative stress in the pancreatic β-cells^15,42,43^. Interestingly, the results showed that the prepared CG-, CG/G-, and G-microcapsules can perfectly inhibit the α-amylase and α-glucosidase activities by 65, 68, 60 and 74, 78, and 70%, respectively, compared to the non-capsulated CSP. This finding can be explained by the improved antioxidant activity of the generated microcapsules as well as the coating materials G and CG may have an inhibitory impact on the examined enzymes. Likewise, both the encapsulated and non-encapsulated piper betel polyphenols were able to inhibit the α-amylase and α-glucosidase activities significantly^19^.

### Antibacterial properties

The antibacterial activity of the produced microcapsules compared to CSP was evaluated against human pathogenic *E. coli* and *S. aureus* bacteria (Table 3). The CG-, CG/G-, and G-microcapsules revealed significantly greater (*p* < 0.01) antibacterial capacity against the investigated bacteria, with low MBC (0.36–0.68 mg ml^−1^) as compared to CSP (0.53–0.74 mg ml^−1^) and Gentamicin (0.7–1.1 mg ml^−1^) (Table 3). This observation suggests that encapsulating the CSP with either CG or G or with both, enhanced the antibacterial activity. This enhancement may be due to the significant increase in antioxidant activity of the generated microcapsules. Additionally, the coating materials G and CG may have an antibacterial effect on the examined pathogenic bacteria. Some phenolic compounds in CSP release H_2_O_2_, which damages bacterial cell membranes and prevents bacteria from growing^42,44^. Further, the micro-sizes of the generated capsules essentially assist in accelerating bacterial cell intake, interfering with protein synthesis, and increasing bacterial cell membrane damage^45^. Some capsules, like CEO, CS/GTO, and S/GG demonstrated better antibacterial activity in comparison to their non-encapsulated extracts^9,24,46^.

**Table 3:** Antibacterial activity of CSP and the prepared microcapsules. Gentamicin is a positive control.

Sample	Bacterial strain MBC (mg/ml)	
	*S. aureus*	*E. coli*
CSP	0.53 ± 0.02^a^	0.74 ± 0.03^a^
CG	0.40 ± 0.02^b^	0.66 ± 0.01^b^
CG/G	0.36 ± 0.01^c^	0.60 ± 0.02^c^
G	0.42 ± 0.01^b^	0.68 ± 0.02^b^
Gentamicin	0.7 ± 0.31^d^	1.1 ± 0.03^d^

Values are provided as means ± SD (n = 4). Different letters in the same column are statistically different at (*P* < 0.01).

CSP, Chia 7-day sprout phenolic extract; CG, Chia gum-microencapsulate; CG/G, Chia gum-gelatin microencapsulate; G, gelatin-microencapsulate; MBC, minimum bactericidal concentration.

## Conclusion

In this work, CSP-phenolic compounds were efficiently encapsulated using the coating materials (CG, G, and their mixture) and a freeze-drying strategy. The three prepared microcapsules retained the most phenolic content, increased antioxidant activity, and great encapsulation efficiency. They demonstrated the efficient bioavailability of CSP-phenolics in simulated intestine fluid. They also displayed long-term storage at 40 °C, good morphological features, and potent interaction between coating materials and CSP-phenolic compound functional groups. In addition, the prepared microcapsules showed stronger antibacterial and antidiabetic activities than the non-capsulated CSP. Thus, the prepared CSP microcapsules can be applied to different food models and used as antidiabetic and antibacterial agents.

### Future challenges and prospects

The prepared CSP microcapsules can be added to some food materials to utilize their high amount of antioxidant phenolic compounds on a large industrial scale. They can also be tested in some pharmaceutical formulations as antimicrobial and anti-diabetic agents. In addition, this study showed the vital role of chia gum in the microencapsulation of phenolic compounds. The plant-gum extraction is simple and cost-effective compared to other polymers. Therefore, additional studies are required to fully understand how gums are used to encapsulate phenolic compounds.

## Data Availability

The datasets generated during and/or analyzed during the current study are available from the corresponding author on reasonable request.
